# Ovatifolin Purified from *Leptocarpha rivularis* Induces Cell Death in A375 and A2058 Melanoma Cancer Cells

**DOI:** 10.3390/antiox14121392

**Published:** 2025-11-21

**Authors:** Viviana Burgos, Nicole Cortez, Rocío Aguilera-Paillán, Sofía Bravo-Bouchat, Bernd Schmidt, Eric Sperlich, Rebeca Pérez, Nelia M. Rodriguez, Leandro Ortiz, Jaime R. Cabrera-Pardo, Cecilia Villegas, Cristian Paz

**Affiliations:** 1 Escuela de Tecnología Médica, Facultad de Salud, Universidad Santo Tomás, Temuco 4780000, Chile; vburgos7@santotomas.cl; 2 Laboratory of Natural Products & Drug Discovery, Center CEBIM, Department of Basic Sciences, Faculty of Medicine, Universidad de La Frontera, Temuco 4780000, Chile; n.cortez01@ufromail.cl (N.C.); rocio.aguilera240@gmail.com (R.A.-P.); sofibrabou@gmail.com (S.B.-B.); 3 Institut für Chemie, Universität Potsdam, Karl-Liebknecht-Str. 24–25, D-14476 Potsdam, Germany; bernd.schmidt@uni-potsdam.de (B.S.); eric.sperlich@uni-potsdam.de (E.S.); 4 Carrera de Química y Farmacia, Facultad de Ciencias de la Salud, Universidad Autónoma de Chile, Avenida Alemania 01090, Temuco 4780000, Chile; rebeca.perez@cloud.uautonoma.cl (R.P.); nelia.roodriguez@uautonoma.cl (N.M.R.); 5 Instituto de Ciencias Químicas, Facultad de Ciencias, Universidad Austral de Chile, Valdivia 5110566, Chile; leandro.ortiz@uach.cl; 6 Laboratorio de Química Aplicada y Sustentable (LabQAS), Departamento de Química, Universidad del Bío-Bío, Avenida Collao 1202, Concepcion 4051381, Chile; jacabrera@ubiobio.cl; 7 Departamento de Ciencias Biológicas y Químicas, Facultad de Recursos Naturales, Universidad Católica de Temuco, Rudecindo Ortega, Temuco 4780000, Chile; cecilia.villegas@uct.cl

**Keywords:** *Leptocarpha rivularis*, ovatifolin, germacrene sesquiterpenoid, melanoma cancer cells

## Abstract

Skin cancer is increasing worldwide, with melanoma being its most aggressive and lethal form due to its high metastatic potential. Despite therapeutic advances, drug resistance remains a challenge, highlighting the need to explore new anticancer agents. *Leptocarpha rivularis* is a native plant of Chile, locally called “Palo negro”, and is traditionally used in medicine by the Mapuche people. *L. rivularis* produces bioactive germacrene sesquiterpenoids with cytotoxic, antioxidant, anti-inflammatory and anti-angiogenic properties. This study reports for the first time the isolation of ovatifolin from aerial parts of *L. rivularis* and its identification by NMR and X-ray diffraction, together with its antiproliferative activity against two melanoma cell lines. The results show that ovatifolin has cytotoxic activity against the cell lines A2058 and A375, with an IC_50_ of 27.6 (90.2 µM) and 18.4 µg/mL (60.1 µM), respectively, evaluated by live-cell IncuCyte^®^ analysis. Moreover, ovatifolin arrests colony formation in a clonogenic assay, with an IC_50_ of 3.26 (10.6 μM) and 3.65 µg/mL (11.9 μM) in these same cell lines. Therefore, ovatifolin increased intracellular ROS and decreased the mitochondrial membrane potential (ΔΨ m). Cell death studies using Annexin V showed that its cytotoxic activity is partially caused by non-specific apoptosis, which was corroborated by the caspase inhibitor Z-VAD with an incomplete recovery of the cell death process.

## 1. Introduction

Skin cancer is the sixth most common type of cancer worldwide. Exposure to UV rays, a weakened immune system, family history, etc., may be the cause of cancer development. The skin is composed of three types of cells: basal cell, squamous cell and melanocytes [1]. There are three main types of skin cancer. Basal cell carcinoma, which originates in the basal cells of the epidermis, is the most common type of skin cancer. It grows slowly and rarely spreads to other parts of the body. There is also spinocellular (epidermoid) carcinoma, which develops in the squamous cells of the epidermis and is the second most common type of non-melanoma skin cancer, which, if not treated in time, can spread to other tissues and grow rapidly. Finally, melanoma originates in melanocytes, pigmented cells located in the basal layer of the epidermis; this is the most aggressive and dangerous type of skin cancer due to its high capacity to metastasize and spread to other organs rapidly [2]. Cutaneous melanoma is the most lethal primary cutaneous neoplasm, accounting for 80% of skin cancer deaths and 1.7% of cancer diagnoses worldwide, being the fifth most common cancer in the USA [3]. The incidence of melanoma has been steadily rising in developed countries, particularly among light-skinned populations, with an increase of approximately 320% in the United States since 1975 [4]. Despite significant advances in immunotherapy and targeted therapy, many patients often develop resistance to treatment, making it necessary to search for new anticancer agents capable of combating this type of neoplasm [5].

Natural products and their structural analogs have historically contributed significantly to pharmacotherapy, especially for the treatment of cancer [6]. However, the pharmacological study of bioactive natural compounds requires their purification and structure analysis [7]. *Leptocarpha rivularis* commonly known as “Palo negro”, is a plant native to South America belonging to the Asteraceae family, which is distributed worldwide with the largest number of species [8,9]. This plant grows in humid and sunny soils, reaching two meters in height, and is traditionally used by the Mapuche people to treat gastrointestinal disorders. Previous phytochemical studies of *L. rivularis* have shown that leaves, bark and flowers contain a wide variety of bioactive compounds with antioxidant, anti-inflammatory, antimicrobial, hypoglycemic and anticarcinogenic activity [9,10,11,12]. Its floral extracts contain a diversity of molecules, including the sesquiterpene lactone leptocarpine, with cytotoxic effects against different types of cancer. In cervical cancer cells (HeLa), leptocarpine or *L. rivularis* extracts show a significant decrease in cell viability and decrease the expression of IL-6, MMP2 and genes associated with carcinogenic activity [13]. In addition, flower extracts of *L. rivularis* reduce proliferation, survival and propagation of gastric cancer cells AGS and MKN-45, together with the vasculogenic capacity, in human endothelial cells EA.hy926, inhibiting angiogenesis [13]. However, the phytochemical composition of the plant is not fully known, nor is its bioactivity against melanoma cancer cells. This study reports the isolation of ovatifolin from aerial parts of *L. rivularis*, structural analysis by single crystal X-ray diffraction and NMR spectroscopy, and the antiproliferative activity of this germacrane against the melanoma cancer cell lines A2058 and A375.

## 2. Materials and Methods

### 2.1. Isolation and Structure Elucidation of Ovatifolin

Ovatifolin was isolated from the aerial parts (6.8 kg) of *Leptocarpha rivularis* collected in Villarica, IX Region of Chile, in January 2024. A voucher specimen was kept in the herbarium with the code LR-2024-01. The leaves were washed and dried at 50 °C and then were ground to obtain a size of less than 1 mm to facilitate the extraction process. The powdered material was extracted by maceration and sonication, using ethyl acetate (EtOAc) in a 1:10 ratio for 2 days at room temperature, with six 10 min sonication cycles (20 kHz, 150 W) at 40 °C, with 1 h intervals between each cycle. The organic phase was evaporated under vacuum at 40 °C and 200 mbar, generating a total organic extract of 120 g. This extraction was performed 3 times to ensure a full extraction. The total extract was fractionated by affinity chromatography (CC) on silica gel (Merck 60). The fractionation was performed using a stepwise gradient elution with the following specific conditions: starting with 100% hexane, followed by hexane:EtOAc mixtures in increasing polarity (9:1, 8:2, 7:3, 6:4, 1:1, 3:7, 2:8, 1:9, and finally 100% EtOAc). Each step used 2 L of solvent mixture, giving ten fractions with different molecular compositions evaluated by thin layer chromatography (TLC). Fraction F8 (54.112 g) was further purified by exclusion chromatography in Sephadex LH20 using EtOAc:methanol 1:1 *v*/*v* as the mobile phase and CC in silica gel Merck 40 CC with hexane:EtOAc 1:1 *v*/*v*, giving a white solid, which was recrystallized in EtOAc at 4 °C. Thus, 45 mg of colorless crystals was obtained.

The structural analysis was performed by NMR spectroscopy in CDCl_3_ using a Bruker Avance NEO 500 spectrometer (Bruker Biospin GmbH, Rheinstetten, Germany). NMR spectra were recorded at 500 MHz for ^1^H and 125 MHz for ^13^C. Signal assignments are based on the 2D-NMR experiments H,H-COSY, NOESY, HSQC, and HMBC. Copies of all spectra, full signal assignments, and comparison with reference data available in the literature are provided in the Supporting Information (Table S2 and Figures S7–S11).

The crystal structure was determined by single-crystal structure analysis. Suitable single crystals were selected using a Leica M205C light microscope and separated with oil. X-ray crystal structure analysis was performed on a Stadivari diffractometer (Stoe) with monochromated Mo-*K*α radiation (λ = 0.71073 Å). Data correction was performed using the program X-Area [14]. The structures were solved by direct methods and refined against *F*^2^ on all data by full-matrix least-squares using the SHELX suite of programs [15]. All non-hydrogen atoms were refined anisotropically; the hydrogen atoms were placed on calculated positions. Table 1 was created using FinalCif. (FinalCif. https://dkratzert.de/finalcif.html (accessed on 1 July 2025) The crystal structure was visualized with Mercury [16]. The data (CCDC 2388201) can be obtained free of charge from The Cambridge Crystallographic Data Centre, http://www.ccdc.cam.ac.uk (accessed on 1 July 2025). More information is given in the Supplementary Materials (Table S1 and Figures S1–S6). 

### 2.2. Cell Culture

Human melanoma cells, A2058 and A375, were obtained from the American Type Culture Collection (ATCC) from Merck (Santiago de Chile, A375 Acc No: 88113005 and A2058 Acc No: 91100402) and cultured in RPMI-1640 medium (Cytiva, Marlborough, MA, USA) and DMEM, respectively, both supplemented with 10% fetal bovine serum (FBS) (Cytiva, Marlborough, MA, USA), 100 U/mL penicillin, and 0.1 mg/mL streptomycin (Cytiva, Marlborough, MA, USA) at 37 °C in a humidified atmosphere with 5% CO_2_. A2058 is a cell exhibiting epithelial morphology isolated from the skin of a white, 43-year-old male patient with melanoma. A375 is a cell line exhibiting epithelial morphology that was isolated from the skin of a 54-year-old, female patient with malignant melanoma.

### 2.3. Cell Viability Analysis

CellTiter 96^®^ Aqueous One Solution Cell Proliferation Assay (MTS) (Promega, Madison, WI, USA) was used to determine the toxic effect of ovatifolin on A375 and A2058 cells. The MTS assay is based on the conversion of a tetrazolium salt into a colored aqueous soluble formazan product by mitochondrial activity from viable cells. The amount of formazan produced by dehydrogenase enzymes is directly proportional to the number of living cells in culture. The viability assays were performed according to the manufacturer’s protocols. Briefly, A375 and A2058 cells were placed into 96-well plates (5 × 10^3^ cells per well) in 100 μL and incubated at 37 °C. Then, cells were exposed to different concentrations of ovatifolin: 0.1, 1, 2.5, 5, 10, 50 and 100 μg/mL (0.3, 3.3, 8.2, 16.3, 32.7, 81.7 and 326.8 µM). After 24 h of incubation, 20 μL of MTS reagent was added to each well, followed by a 4 h incubation at 37 °C. The absorbance was measured by a microplate reader (VICTOR NivoTM Perkin Elmer, Waltham, MA, USA) at 490 nm. Results were expressed as the percentage of viability relative to the control.

### 2.4. IncuCyte^®^ Real-Time Cell Death Assay

To assess the cytotoxic activity of ovatifolin in real time, A-2058 and A-375 cells were cultured in 96-well plates at a density of 5 × 10^3^ cells per well. Cells were then exposed to a gradient of ovatifolin concentrations of 0.1, 1, 2.5, 5, 10, 50, and 100 μg/mL (0.3, 3.3, 8.2, 16.3, 32.7, 81.7 and 326.8 µM) prepared in RPMI-1640 or DMEM, depending on the cell line, each supplemented with 0.1% (*v*/*v*) DMSO. The assays were carried out using the IncuCyte^®^ S3 live-cell imaging (Bohemia, NY, USA) in combination with the cell-impermeant dye Sytox Green (30 nM; Invitrogen™, #S7020, Carlsbad, CA, USA) as an indicator of membrane integrity loss [17]. Fluorescent signals corresponding to dead cells were monitored and quantified automatically at hourly intervals for a total duration of 48 h. From these data, dose–response curves were generated and IC_50_ values were determined using GraphPad Prism 8.0 software. Furthermore, cell confluence, expressed as the percentage of image area covered by adherent cells, was quantified with IncuCyte^®^ v2019B software at the Advanced Microscopy Center (CMA Biobío, University of Concepción, Concepción, Chile).

### 2.5. Clonogenic Assays

Determination of the antiproliferative activity of ovatifolin at different concentrations was confirmed by a clonogenic assay; cells were grown at a density of 1000 per well in a 12-well plate and then incubated with increasing concentrations of ovatifolin (0.1, 1, 5, 10, 25 and 50 μg/mL (0.3, 3.3, 16.3, 32.7, 81.7 and 163.4 µM)) in RPMI-1640 medium for A2058 cells and DMEM for A375 cells, both with 10% SFB. Following a 3 h exposure, the compound was withdrawn and the cells were maintained for an additional 11 days in culture with regular medium replacement. At the end of this period, colonies were fixed in 80% ethanol for 15 min at room temperature, stained with 0.5% crystal violet for 20 min, and subsequently imaged. Quantification of the total colony area under each condition was performed using ImageJ software V.1.49 (NIH, USA) [18,19].

### 2.6. Cell Apoptosis Detection by Flow Cytometry

Apoptosis was evaluated using the Annexin V-Alexa Fluor™ 488 Apoptosis Detection Kit combined with propidium iodide (cat# V13241, Invitrogen™, Invitrogen, Carlsbad, CA, USA), following the protocol described by Lakshmanan and Batra [20]. A375 cells were plated at a density of 5 × 10^5^ cells/mL in 60 mm culture dishes and allowed to adhere for 24 h. Thereafter, cells were exposed for 24 h to ovatifolin 18.4 μg/mL (60.1 μM), camptothecin (0.4 μM, positive control), or 0.1% DMSO (vehicle control). After treatment, cells were detached with trypsin, collected, and incubated with Annexin V/PI for 15 min at room temperature under dark conditions. Samples were then analyzed using a FACSCanto II flow cytometer (Becton Dickinson, Franklin Lakes, NJ, USA) with excitation at 488 nm and emission at 530 nm. Cell populations were discriminated according to staining pattern: early apoptotic (Annexin V-positive/PI-negative), late apoptotic or necrotic (Annexin V-positive/PI-positive), and necrotic (Annexin V-negative/PI-positive).

### 2.7. ROS Measurement

A2058 and A375 cells were seeded at a density of 15,000 cells per well in black, flat-bottom 96-well plates and maintained at 37 °C under 5% CO_2_ in phenol red-free RPMI-1640 and DMEM, respectively. After a 24 h incubation period, cells were loaded with 5 μM 2,7-dichlorodihydrofluorescein diacetate (H_2_DCFDA; #5935, Tocris Bioscience, Bristol, UK) for 30 min, following the manufacturer’s instructions and referencing previously described procedures [21], and then treated with ovatifolin 18.4 μg/mL (60.1 μM); H_2_O_2_ (330 μM) was used as a positive control. DMSO 0.1% was used as control. Fluorescence was then recorded every hour for 5 h using the VICTOR Nivo™ (PerkinElmer, Waltham, MA, USA) microplate reader at ex 495 nm/em 520 nm.

### 2.8. Mitochondrial Membrane Potential Analysis

This analysis was performed in real time using the IncuCyte^®^ S3 live-cell analysis system (Bohemia, NY, USA) system, with JC-1 as a mitochondrial membrane potential indicator, adjusting the manufacturer’s instructions to the protocol recommended by the provider and using a mitochondrial membrane potential disrupter, CCCP (carbonyl cyanide 3-chlorophenylhydrazone), as a positive control (50 μM). The JC-1 probe is a lipophilic dye that accumulates in mitochondria depending on membrane potential. Under normal conditions, JC-1 will aggregate in the form of aggregates that emit red fluorescence (R). In contrast, if the membrane potential is low, JC-1 will remain in the form of monomers that emit green fluorescence (G). A high R/G value suggests that the mitochondrial membrane potential is normal or more active. On the other hand, a low R/G value indicates that the mitochondrial membrane potential is low, so depolarization of the mitochondrial membrane can be assumed. A2058 and A375 cells (5 × 10^3^ cells/well) were seeded in 96-well plates for 6 h. JC-1 stain (2 μM final concentration) was then added and the cells were incubated at 37 °C, 5% CO_2_, for 15 to 30 min. Afterward, A2058 and A375 cells were treated with 10 y 18,4 µg/mL (32.7 and 60.1 μM) of ovatifolin, respectively, vehicle (DMSO 0.1%) and positive control CCCP (50 µM). Plates were kept incubated and fluorescence intensity was recorded at 2 h at 514/529 and 590 nm. Images were processed using IncuCyte^®^ S3 live-cell analysis system (Bohemia, NY, USA) software (v.2019B).

## 3. Results

### 3.1. Chemical Characterization of Ovatifolin from Leptocarpha rivularis

Ovatifolin, C_17_H_22_O_5_ (Figure 1), is a germacrane-type sesquiterpene lactone isolated from *Leptocarpha rivularis* in a 0.00066% yield from fresh aerial parts of the plant; it was identified by XRD (Figure 1 and Figures S1–S6) and 1D and 2D NMR at 500 MHz (Table 1; full NMR results are provided in Figures S7–S11).

### 3.2. Ovatifolin Shows Cytotoxic Effects Against A2058 and A375 Cells Lines

**Figure 2:**
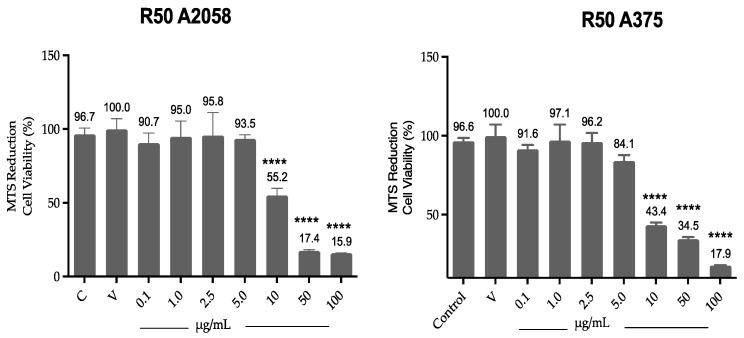
Effect of ovatifolin on the cell viability of A375 and A2058. Cell viability was assessed by MTS assay and compared to control (cells without treatment). The data are expressed as mean ± standard deviation. ANOVA, Dunnett’s Multiple Comparison Test (**** = *p* < 0.0001 vs. Control).

Cell viability results by MTS (Figure 2) show that ovatifolin significantly reduces viability from a concentration of 10 µg/mL (32.7 µM) in both A2058 and A375 cell lines, with viabilities of 55.1% and 44.3%, respectively, increasing with higher concentrations.

**Figure 3:**
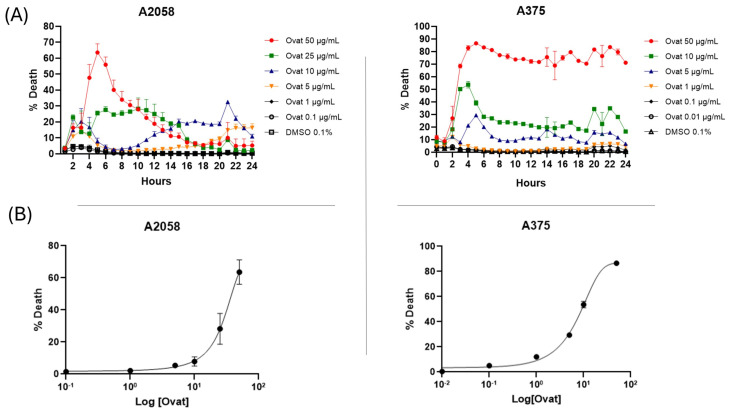
(**A**) Realtime plots obtained by IncuCyte^®^ live-cell analysis system used to study cell death measurements (24 h, %) in A2058 and A375 cells exposed to 0.01–50 μg/mL (0.03–163.4 µM) of ovatifolin. Data are presented as mean ± SD (*n* = 3). (**B**) Dose–response curves evaluated using IncuCyte^®^ at 24 h. The percentage of cell death is plotted as a function of the logarithmic concentration of the compounds, with the IC_50_ value for ovatifolin.

Real-time cytotoxicity evaluation using the IncuCyte live cell analysis system, as shown in Figure 3A, indicated that ovatifolin has cytotoxic effects against advanced melanoma cell lines. Ovatifolin at a concentration of 50 µg/mL (163.4 µM) showed rapid cytotoxic action against both A2058 and A375 cell lines, reaching over 60% and 80% death, respectively, at 5 h. However, the death marker Sytox starts to quench after 5 h, mostly in the A2058 cell line at higher concentrations, which could be associated with the fact that the compound may accelerate cellular DNA degradation. Ovatifolin at concentrations of 25 (81.7 µM) and 10 µg/mL (32.7 µM) showed less cytotoxicity, but there was still cell death in both cell lines. However, lower concentrations of ovatifolin of 0.01–5 µg/mL (0.03–16.3 µM) did not cause cell death.

**Figure 4:**
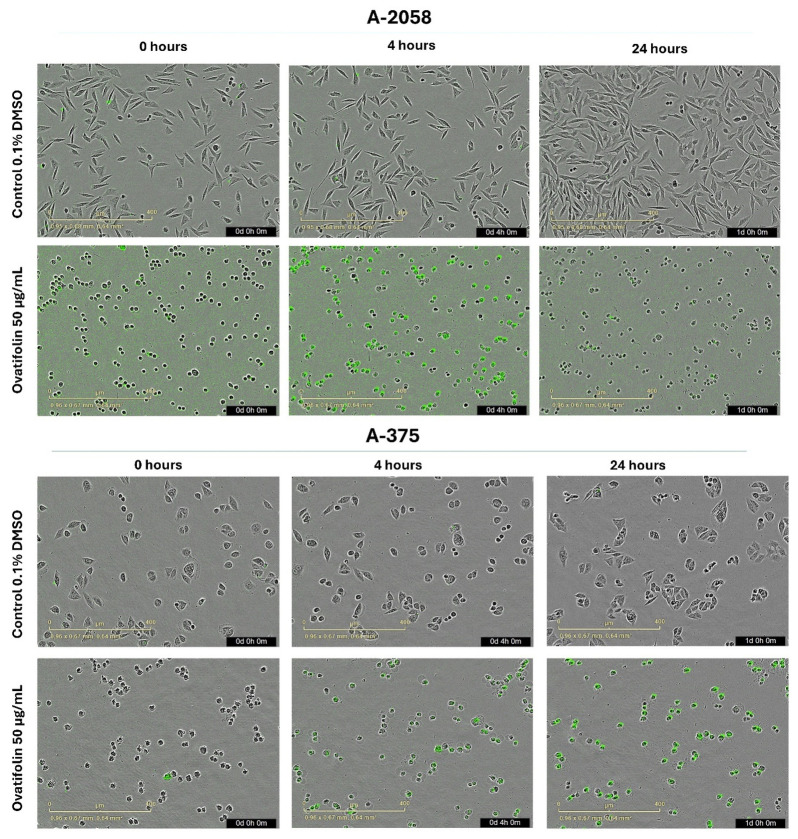
Microscopy by IncuCyte^®^ live-cell analysis system used to study images of Sytox Green-stained A2058 and A375 cells treated with ovatifolin 50 μg/mL (163.4 µM); observations at 0, 4 h and 24 h after the end of treatment.

Microscopy images (Figure 4) obtained for each hour throughout the experiment revealed that treatment with ovatifolin 50 μg/mL (163.4 µM) induced a progressive increase in the Sytox signal (green) up to 5 h for both lines, indicating an increase in the permeability of the marker characteristic of cell death. After 5 h, one of the A2058 lines showed a decrease in the signal, while in the A375 line, it remained relatively constant, which, as mentioned above, could be associated with a rapid degradation of the cellular genetic material. However, ovatifolin showed higher potency against A2058 than A375, showing that it affected A2058 cells from time 0, as seen in Figure 4.

Both A2058 and A375 cell lines exhibited a hallmark apoptotic morphology, including cell shrinkage, formation of apoptotic bodies, and loss of monolayer integrity immediately upon treatment with high concentrations of ovatifolin (50 µg/mL (163.4 µM)).

The dose–response curve plots shown in Figure 3B show that ovatifolin has the most potent cytotoxic activity against the A375 cell line, with an IC_50_ of 18.4 μg/mL (60.1 µM), while it was shown to have a lower cytotoxic effect against the A2058 line, with an IC_50_ of 27.6 μg/mL (90.2 µM).

### 3.3. Ovatifolin Reduces the Proliferative Activity of A2058 and A375 Cells

**Figure 5:**
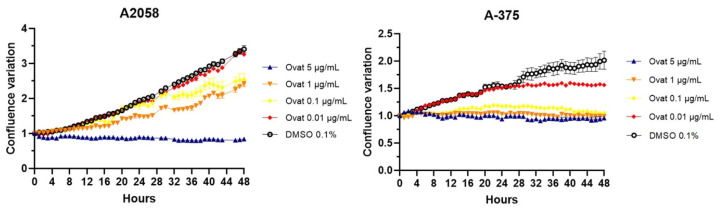
Time–course analysis of cell confluence in A2058 and A375 cell lines treated with ovatifolin at 0.01, 0.1, 1 and 5 μg/mL (0.03, 0.3, 3.3 and 16.3 µM), by IncuCyte^®^ live-cell analysis system. Data are presented as mean ± SD (*n* = 3).

Real-time confluence measurements using IncuCyte^®^ (Figure 5) showed that ovatifolin inhibits proliferation at non-toxic concentrations (5 μg/mL (16.3 µM)) after 48 h compared to the untreated control (*p* < 0.05). In this regard, it was observed that the cytostatic effects are more pronounced in A375 cells, where ovatifolin, even at low concentrations (0.01 µg/mL (0.03 µM)) manages to inhibit cell proliferation by almost at 100%. Ovatifolin at low concentrations exhibited cytostatic effects in both A2058 and A375 melanoma cell lines.

**Figure 6:**
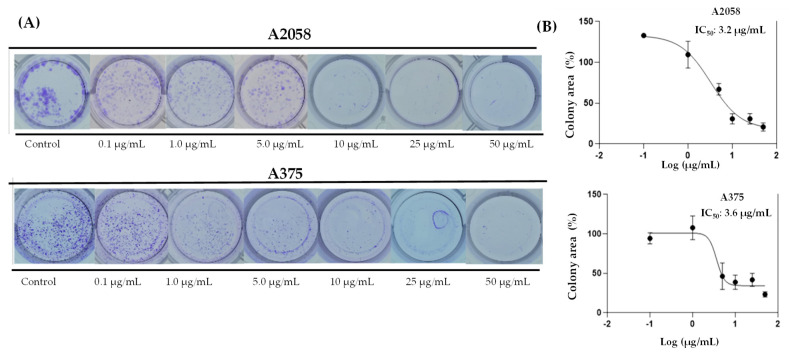
Colony formation assays on A2058 and A375 cell lines exposed to ovatifolin at different concentrations (0.1 to 50 µg/mL (0.3 to 163.0 µM)) for 3 h. (**A**) Representative images of colonies stained with crystal violet after 11 days of culture following germacrane exposure. (**B**) IC_50_ values for colony formation were determined. The percentage of colony area was quantified using ImageJ V.1.49 (NIH, USA), enabling precise analysis of treatment effects on long-term cell viability and proliferation.

In support of these results, it was tested whether prolonged exposure to ovatifolin would prevent colony formation of advanced A375 and A2058 cells. Figure 6 shows the results of the 11-day clonogenic assays, which shows that ovatifolin effectively inhibited colony formation in both cell lines. The IC_50_ values were 3.3 µg/mL (10.8 µM) in A2058 and 3.7 (12.1 µM) µg/mL in A375.

### 3.4. Ovatifolin Exhibits Proapoptotic and Necrotic Properties in A375 Melanoma Cells

**Figure 7:**
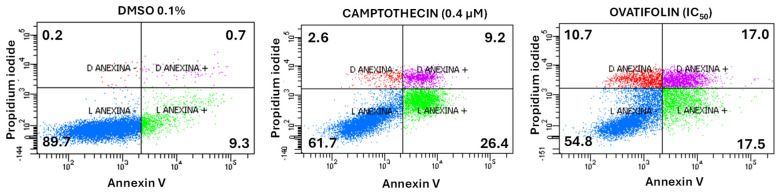
Flow cytometry analysis of cell death induction by ovatifolin at 18.4 μg/mL (60.1 µM) in A375 melanoma cancer cells. Annexin V and propidium iodide (PI) were used to distinguish between viable cells (Annexin V−/PI−), early apoptotic cells (Annexin V+/PI−), late apoptotic or secondary necrotic cells (Annexin V+/PI+), and primary necrotic cells (Annexin V−/PI+). Camptothecin (0.4 μM) was used as a positive control, while 0.1% DMSO served as a negative control. Treatment with ovatifolin reduced the viability of A375 cells and increased apoptosis levels. The percentage of cells in each state is shown in the respective quadrants.

Annexin V and propidium iodide (PI) assay was performed to determinate the cytotoxic mechanism of ovatifolin. The apoptosis/necrosis assay showed that ovatifolin significantly induced apoptosis at 18.4 μg/mL (60.1 µM) after 24 h (Figure 7), similarly to camptothecin (a positive control that induces apoptosis). In treated cells, ovatifolin produced a 54.8% decrease in viable cells, with an increase in late and early apoptosis in equal proportion (17%). However, compared to camptothecin, ovatifolin induced primary necrosis in 10.7% (Figure 7) of cells, suggesting that ovatifolin is cytotoxic in an unspecific manner, through a combination of cell death types.

**Figure 8:**
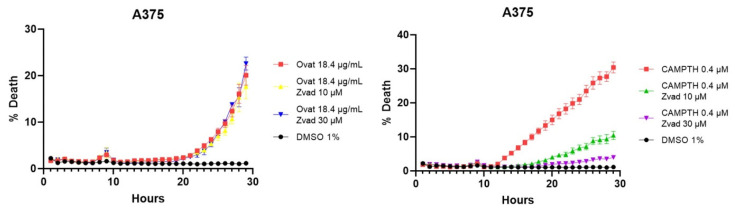
Cell death curves caused by ovatifolin and camptothecin as a positive control in melanoma lines A2058 and A375, with the Z-VAD caspase inhibitor to corroborate specific death by apoptosis.

To confirm these results, tests were performed with an apoptosis inhibitor, more specifically the Z-VAD caspase inhibitor. These tests corroborate that cell death is not mainly caused by apoptosis, since, as shown in Figure 8, when adding ovatifolin together with the inhibitor, the percentage of cell death does not decrease in A375 cells, while it does in the case of the positive control camptothecin.

### 3.5. Ovatifolin Increases Cellular ROS Production and Decreases Mitochondrial Membrane Potential in Melanoma Cancer Cells

**Figure 9:**
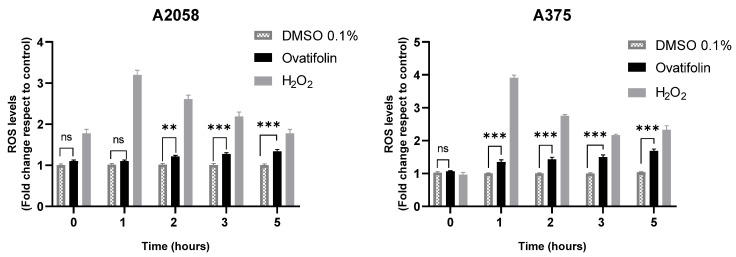
Quantification of ROS by H_2_DCFDA probe in A2058 and A375 melanoma cell lines. Significant differences between ovatifolin and the control are marked (** *p* < 0.01, *** *p* < 0.001); no significant difference is indicated as “ns”. H_2_O_2_, used as a positive control, led to a significant increase at all measured times.

The production of reactive oxygen species (ROS) induced by ovatifolin was evaluated in A2058 and A375 melanoma cells using the fluorescent probe H_2_DCFDA. Figure 9 shows that, in both cell lines, treatment with ovatifolin 18.4 μg/mL (60.1 µM) increased intracellular ROS levels with respect to time 0, as observed from 2 h in A2058 (** *p* < 0.01) and from 1 h in A375 (*** *p* < 0.001). This effect persisted and intensified over time, reaching highly significant differences at 5 h (*** *p* < 0.001 in A2058; *** *p* < 0.001 in A375). Hydrogen peroxide (H_2_O_2_) was used as a positive control and led to a marked increase in ROS levels at all time points. These results indicate that ovatifolin promotes ROS accumulation in a time-dependent manner, suggesting a possible oxidative mechanism of action in melanoma cells.

**Figure 10:**
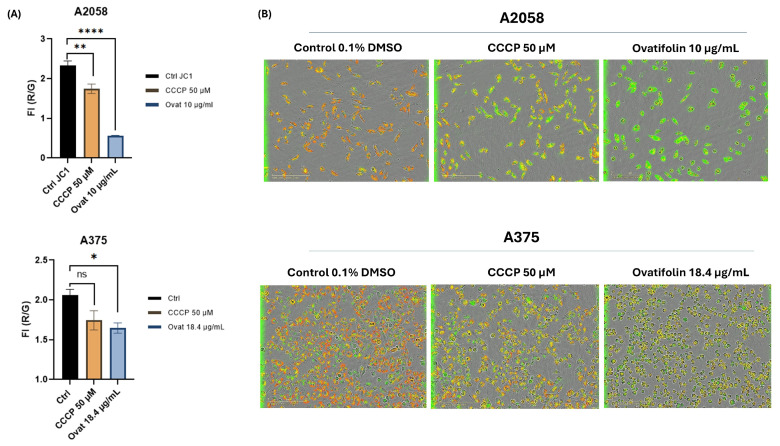
Effects of ovatifolin on the mitochondrial membrane potential of A2058 and A375 melanoma cells. (**A**) Quantification of reduction in ΔΨ m following treatment at 2 h with ovatifolin 27.6 and 18.4 μg/mL (90.2 and 60.1 µM) for A2058 and A375 cell lines, respectively, measured by loss in red/green fluorescence compared to the control (* *p* < 0.05, ** *p* < 0.01, **** *p* < 0.001); “ns”: no significant differences. Data are presented as the means of three replicates. (**B**) Microscopy images of fluorescence with JC-1 under different conditions at time 2 h of the experiment (6 h).

Regarding mitochondrial function, depolarization kinetic studies by time course analysis of live IncuCyte^®^ cells showed that ovatifolin caused a decrease in ΔΨ m in the A2048 and A375 cell lines compared to the control at 2 h of treatment (Figure 10A). These results were confirmed by microscopic images on IncuCyte^®^, showing a decrease in red fluorescence and an increase in green fluorescence, indicative of the loss of ΔΨ m (Figure 10B). These results highlight the rapidity of the cytotoxic action of ovatifolin on melanoma cell lines.

## 4. Discussion

This study reports the first isolation and structural characterization of ovatifolin from *Leptocarpha rivularis*. Previous phytochemical investigations documented this germacrane sesquiterpene lactone in several Asteraceae species, including *Podanthus ovatifolius* [22,23], *P. mitiqui* [24,25], *Inula germanica* [26,27], *Greenmaniella resinosa* [28], *I. salsoloides* [29,30], *Blainvillea latifolia* [28], and *Calea uniflora* [20]. While biological studies have primarily focused on antioxidant and anti-inflammatory properties [25], with limited reports of activity against Leishmania amazonensis amastigotes [20], comprehensive cytotoxicity assessments remain scarce. The present investigation demonstrates that ovatifolin exhibits concentration-dependent effects against human melanoma cell lines A2058 and A375, with IC_50_ values of 27.6 µg/mL (90.2 µM) and 18.4 µg/mL (60.1 µM), respectively (Figure 3). Notably, A375 cells demonstrated enhanced sensitivity to antiproliferative effects at sub-cytotoxic concentrations, as evidenced by a reduced confluence at 5 µg/mL (16.3 µM) after 48 h (Figure 5). Clonogenic assays further revealed potent inhibition of long-term colony formation, with IC_50_ values of 3.3 µg/mL (10.8 µM) in A2058 and 3.7 µg/mL (12.1 µM) in A375 after 11-day culture (Figure 6), indicating distinct cytostatic properties at concentrations approximately 7-fold lower than acute cytotoxic thresholds.

Comparatively, the structurally related sesquiterpene lactone isocostunolide exhibits higher potency against A2058 cells (IC_50_ 3.2 µg/mL, ~8-fold more potent than ovatifolin) [31], suggesting that structure–activity relationships within this compound class warrant systematic investigation. Similarly, inuviscolide and tomentosin from Inula viscosa induce growth arrest in multiple melanoma lines (SK-28, 624 mel, 1363 mel) through G2/M cell cycle blockade accompanied by sub-G0 DNA fragmentation, consistent with apoptotic mechanisms [32]. The distinct cytotoxic profiles observed across germacrane derivatives highlight the influence of subtle structural modifications on biological activity and mechanistic pathways. Cytotoxicity analysis by IncuCyte^®^ showed a progressive increase in death up to 5 h after the start of treatment with ovatifolin, following by a fast disruption of the plasma membrane. After the first 5 h of treatment, there is an inhibition of fluorescence emission in both cell lines, but especially in the case of A2058 cells, because SYTOX Green is an asymmetric cationic cyanine dye that is excluded from living cells but can readily penetrate cells with compromised cell membranes. Upon binding to cellular nucleic acids, the dye exhibits a large increase in fluorescence, which is monitored at fluorescein wavelengths [33]. Therefore, the signal loss suggests rapid DNA degradation, potentially indicating genotoxic activity (then move micronucleus reference here). Morphological changes in cell death, such as membrane blebbing and cell shrinkage, were observed even immediately after application of the compound (Figure 5). Previously, compounds similar to ovatifolin have been reported to have genotoxic action, determined by the micronucleus test in bone marrow of male *Mus musculus*, Balb/c mice [34].

Flow cytometric analysis using Annexin V/PI demonstrated that ovatifolin increases the proportion of Annexin V+/PI− cells, although less than observed with camptothecin, an inducer of apoptosis (Figure 7). However, it still increases the proportion of Annexin V+/PI+ and Annexin V−/PI+ cells to a greater extent than camptothecin. This suggests that ovatifolin causes cell death in a nonspecific manner, inducing mixed-mode cell death involving apoptotic, necrotic, and potentially ferroptotic pathways, which also involve exposure to phosphatidylserine. The action of this molecule as an inducer of apoptosis was also ruled out using the Z-vad caspase inhibitor probe, showing that the addition of the inhibitor did not decrease the percentage of cell death caused by ovatifolin, suggesting that cell death is induced by a different mechanism than apoptosis (Figure 8).

Moreover, the cytotoxic activity mechanisms of ovatifolin appear to involve multiple pathways. Ovatifolin treatment increased intracellular ROS levels, although this effect occurred more rapidly in the A375 cell line than in the A2058 line (Figure 9). Concurrently, ovatifolin induced mitochondrial depolarization within 2 h, as demonstrated by decreased ΔΨ m (Figure 10). It has been shown that when reactive oxygen species (ROS) levels exceed the capacity of the antioxidant system, direct damage to DNA, proteins and lipids occurs, triggering mitochondrial membrane permeabilization and activating the cascade leading to cell death [35]. This oxidative damage may trigger the observed cell death, although confirmation requires antioxidant rescue experiments such as Trolox [35]. Related germacranes compounds like scabertopin-7, a sesquiterpene lactone derivative of the germacranan-type scabertopin isolated from *Elephantopus scaber*, induce apoptotic cell death, associated with increased generation of reactive oxygen species (ROS), loss of mitochondrial membrane potential, modulation of Bcl-2 family proteins and caspases-3 and PARP cleavage [36]. Also, erioflorin acetate and erioflorin isolated from *P. mitiqui* possess cytotoxic activity in advanced prostate cancer cell lines 22Rv1 and DU-145, with an IC_50_ of 35.1 µM and 27.3 µM, respectively, for erioflorin acetate, and an IC_50_ of 50.3 µM and 56.5 µM, respectively, for erioflorin. Both compounds increased ROS levels, reduced ΔΨ m and induced apoptosis [35].

Taken together, the results presented in this study reveal for the first time the cytotoxic activity of ovatifolin isolated from *Leptocarpha rivularis* on human melanoma cell lines, suggesting that this sesquiterpene compound possesses a multifactorial mechanism of action involving oxidative stress, mitochondrial dysfunction and non-exclusive apoptotic cell death. The abrupt loss of mitochondrial membrane potential and evidence of genetic damage support the hypothesis of a genotoxic effect associated with this compound, although ovatifolin did not show clear activation of classical caspase-dependent apoptosis pathways.

## 5. Conclusions

In summary, this study reports for the first time the isolation and identification of ovatifolin from *Leptocarpha rivularis*, presenting updated crystallographic and high-resolution NMR data. Moreover, it was demonstrated that ovatifolin has antiproliferative activity against melanoma cells A2058 and A375, but real-time analysis by IncuCyte^®^ showed that ovatifolin produces disruption of plasma membrane with degradation of the genetic material, suggesting genotoxic effects, which is detrimental to possible therapeutic uses. Moreover, the mechanism of cell death is not specific, or at least not to apoptosis. The differential response between A375 and A2058 cell lines treated with ovatifolin at 50 μg/mL after 5 h could be due to the distinct kinetic profiles, likely reflecting inherent differences in cellular metabolism, DNA repair capacity, or drug efflux mechanisms between these melanoma cell lines. A375 cells are derived from malignant melanoma from female patients, while A2058 cells originate from a metastatic melanoma. A2058 has a higher growth rate, consistent with a more invasive and aggressive phenotype due to its faster replication. This could be the reason why ovatifolin showed signal loss in our real-time assay system compared to A375. More studies are necessary to fully determine the molecular mechanisms activated by ovatifolin and to evaluate their efficacy and safety in preclinical models, with a view to the development of new therapeutic strategies based on germacrene sesquiterpenoids.

## Figures and Tables

**Figure 1:**
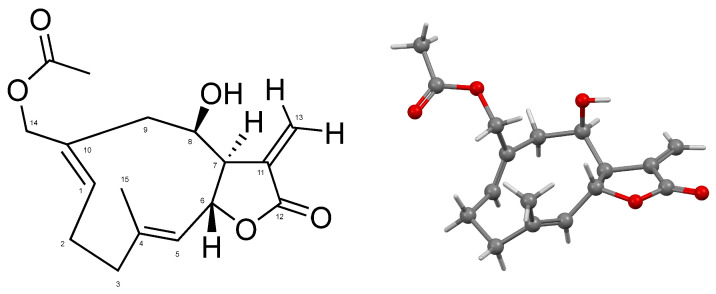
Numbered molecular structure of ovatifolin (**left**). Ball-and-stick representation for the atoms and bonds of ovatifolin in solid state (**right**).

**Table 1:** ^1^H- (500 MHz, CDCl_3_) and ^13^C- (125 MHz, CDCl_3_) NMR data of ovatifolin.

Position	^1^H in δ (ppm)	m (J (Hz))	^13^C in δ (ppm)
1	5.12	dd (12.3, 4.5)	136.5
2	2.42	td (12.4, 5.3)	25.8
	2.29	m	
3	2.36	m	39.1
	2.15	m	
4	--	--	142.2
5	4.84	d (10.2)	127.8
6	5.21	dd (10.2, 8.6)	75.0
7	2.75	dt (8.5, 3.5)	53.7
8	4.58	m	71.3
9	2.94	dd (14.4, 5.4)	42.4
10	--	--	133.2
11	--	--	138.4
12	--	--	170.3
13	6.34 (*Z*-H13)	brd (3.1)	120.5
	5.57 (*E*-H13)	d (3.1)	
14	4.77	d (11.9)	63.2
	4.58	d (11.9)	
15	1.62	d (1.3)	17.0
–C(O)CH_3_	--	--	171.6
–C(O)CH_3_	2.06	s	21.2

brd: broad doublet, d: doublet, dd: doublet of doublets, dt: doublet of triplets, s: singlet

## Data Availability

The crystallographic data are available free of charge from the Cambridge Crystallographic Data Centre (CCDC 2388201). Primary NMR FID files for ovatifolin are openly available via the Zenodo data repository at https://doi.org/10.5281/zenodo.15052366.

## Supplementary Materials

The following supporting information can be downloaded at https://www.mdpi.com/article/10.3390/antiox14121392/s1: Crystallographic details and data; visualization of the crystal structure; ^1^H- and ^13^C-NMR-data of ovatifolin and comparison with literature data; copies of ^1^H-, ^13^C-, COSY, HSQC- and HMBC-NMR-spectra (pdf). Table S1. Crystal data and details of structure refinement for ovatifolin. Figure S1: Molecular structure of ovatifolin with atomic labels. Displacement ellipsoids are shown at the 50% probability level. Figure S2: All hydrogen bonds in ovatifolin with A-H distances of up to 2.6 Å (blue dashed lines) and D-H-A angles (green label) of at least 120°. Figure S3: Zigzag-shaped hydrogen bonding sequence (blue dashed lines) along the a direction in ovatifolin. Figure S4: Packing of different hydrogen bonding chains (blue dashed lines) with a view along the a direction in ovatifolin. Figure S5: Cell view of ovatifolin looking along the crystallographic b axis (with hydrogen bonds as blue dashed lines). Figure S6: Cell view of ovatifolin looking along the crystallographic a axis (with hydrogen bonds as blue dashed lines). Table S2. NMR data of ovatifolin and comparison with literature data for ovatifolin. Figure S7: ^1^H NMR (500 MHz, CDCl_3_) of ovatifolin. Figure S8: ^13^C NMR (125 MHz, CDCl_3_) of ovatifolin. Figure S9: H,H-COSY (500 MHz, CDCl_3_) of ovatifolin. Figure S10: HSQC (500/125 MHz, CDCl_3_) of ovatifolin. Figure S11: HMBC (500/125 MHz, CDCl_3_) of ovatifolin.

## Abbreviations

The following abbreviations are used in this manuscript:

NMR	Nuclear Magnetic Resonance
ROS	Reactive Oxygen Species
IL-6	Interleukin-6
MMP2	Matrix metallopeptidase 2
COSY	Correlation Spectroscopy
NOESY	Nuclear Overhauser Effect Spectroscopy
HSQC	Heteronuclear Single Quantum correlation
HMBC	Heteronuclear Multiple Bond Correlation
ATCC	American Type Culture Collection
FBS	Fetal bovine serum
MTS	Cell Proliferation Assay
DMSO	Dimethyl sulfoxide
H_2_DCFDA	Dichlorodihydrofluorescein diacetate
CCCP	Carbonyl cyanide 3-chlorophenylhydrazone
CDCl_3_	Deuterated chloroform
PI	Propidium iodide
IC_50_	Half-maximal inhibitory concentration
ΔΨ m	Mitochondrial membrane potential
