# Supplementary material for: Ovatifolin Purified from Leptocarpha rivularis Induces Cell Death in A375 and A2058 Melanoma Cancer Cells
Source: Antioxidants (Basel). 2025 Nov 21;14(12):1392. doi: 10.3390/antiox14121392 (PMC12729485; doi:10.3390/antiox14121392)

Supporting Information

Ovatifolin Purified from *Leptocarpha rivularis* Induces Cell Death in A375 and A2058 Melanoma Cancer Cell

Viviana Burgos[1], Nicole Cortez[2], Rocío Aguilera-Paillán[2], Sofía Bravo-Bouchat[2], Bernd Schmidt[3], Eric Sperlich[3], Rebeca Pérez[4], Nelia M. Rodriguez[4], Leandro Ortiz[5], Jaime R. Cabrera-Pardo[6], Cecilia Villegas[7] and Cristian Paz[2*]

[1] Escuela de Tecnología Médica, Facultad de Salud, Universidad Santo Tomás, Temuco 4780000, Chile. vburgos7@santotomas.cl

[2] Laboratory of Natural Products & Drug Discovery, Center CEBIM, Department of Basic Sciences, Faculty of Medicine, Universidad de La Frontera, Temuco 4780000, Chile. n.cortez01@ufromail.cl (N.C.); rocio.aguilera240@gmail.com (R. A-P.); sofibrabou@gmail.com (S.B-B.)

[3] Institut für Chemie, Universität Potsdam, Karl-Liebknecht-Str. 24-25, Potsdam D-14476, Germany. bernd.schmidt@uni-potsdam.de (B.S.); eric.sperlich@uni-potsdam.de (E. S.)

[4] Carrera de Química y Farmacia, Facultad de Ciencias de la Salud, Universidad Autónoma de Chile, Avenida Alemania 01090, Temuco 4780000, Chile. rebeca.perez@cloud.uautonoma.cl; nelia.roodriguez@uautonoma.cl

[5] Instituto de Ciencias Químicas, Facultad de Ciencias, Universidad Austral de Chile, Valdivia 5110566, Chile; leandro.ortiz@uach.cl

[6] Laboratorio de Química Aplicada y Sustentable (LabQAS), Departamento de Química, Universidad del Bío-Bío, Avenida Collao 1202, Concepcion 4051381, Chile; jacabrera@ubiobio.cl

[7] Departamento de Ciencias Biológicas y Químicas, Facultad de Recursos Naturales, Universidad Católica de Temuco, Rudecindo Ortega, Temuco 4780000, Chile. cecilia.villegas@uct.cl

* Correspondence: cristian.paz@ufrontera.cl; Tel.: +56 45 259 2825.

Contents:

A **Single crystal X-ray structure analyses of ovatifolin** **S2**

B **NMR-Spectroscopical analysis of ovatifolin** **S7**

A Single crystal X-ray structure analyses of ovatifolin

1 General details of X-ray structure analysis

The crystal structure of ovatifolin has been published once in the CSD in 1977 and can be found under the CSD code OVATOM.[1] Since the intermolecular interactions of the compound were not discussed in this publication, this information will be provided here.

The crystal structure was determined by single crystal structure analysis. Suitable single crystals were selected using a Leica M205C light microscope and separated with oil. X-ray crystal structure analysis was performed on a Stadivari diffractometer (Stoe) with monochromated Mo-*K*α radiation (λ = 0.71073 Å). The data correction was performed using the program X-Area.[2] The structures were solved by direct methods and refined against F^2 on all data by full-matrix least-squares using the SHELX suite of programs.[3,4] All non-hydrogen atoms were refined anisotropically; the hydrogen atoms were placed on calculated positions. **Table 1** was created using FinalCif.[5] The crystal structure was visualized with Mercury.[6] The data (CCDC 2388201) can be obtained free of charge from The Cambridge Crystallographic Data Centre, http://www.ccdc.cam.ac.uk.

References

(1) Gopalakrishna, E. M.; Watson, W. H.; Hoeneisen, M.; Silva, M. Ovatifolin, a sesquiterpene lactone. *J. Cryst. Mol. Struct.* **1977**, *7*, 49-57.

(2) STOE & Cie GmbH (2018) X-Area. software package for collecting single-crystal data on STOE area-detector diffractometers, for image processing, for the correction and scaling of reflection intensities and for outlier rejection. STOE & Cie GmbH, Darmstadt.

(3) Sheldrick, G. Crystal structure refinement with SHELXL. *Acta Cryst. C* **2015**, *C71*, 3-8.

(4) Sheldrick, G. A short history of SHELX. *Acta Cryst. A* **2008**, *A64*, 112-122.

(5) FinalCif. https://dkratzert.de/finalcif.html.

(6) Macrae, C. F.; Sovago, I.; Cottrell, S. J.; Galek, P. T. A.; McCabe, P.; Pidcock, E.; Platings, M.; Shields, G. P.; Stevens, J. S.; Towler, M.; Wood, P. A. Mercury 4.0: from visualization to analysis, design and prediction. *J. Appl. Cryst.* **2020**, *53*, 226-235.

(7) Hoeneisen, M.; Silva, M.; Bohlmann, F. Sesquiterpene lactones of *Podanthus mitiqui*. *Phytochemistry* **1980**, *19*, 2765-2766.

2 Crystallographic Data:

Table S1. Crystal data and details of structure refinement for ovatifolin.

Compound	**ovatifolin**
CCDC number	2388201
Empirical formula	$C_{17}H_{22}O_5$
Formula weight	384.41
Temperature [K]	297(2)
Crystal system	triclinic
Space group (number)	$P\bar{1}$ (2)
a [Å]	7.1953(3)
b [Å]	11.5458(6)
c [Å]	12.2013(6)
α [°]	77.685(4)
β [°]	84.917(4)
γ [°]	88.162(4)
Volume [Å^3]	986.32(8)
Z	2
ρ_{calc} [gcm^{-3}]	1.294
μ [mm^{-1}]	0.094
F(000)	408
Crystal size [mm^3]	0.600×0.333×0.200
Crystal colour	colorless
Crystal shape	plate
Radiation	Mo K_α (λ=0.71073 Å)
2θ range [°]	6.88 to 49.99 (0.84 Å)
Index ranges	$-8 \leq h \leq 8$<br>$-13 \leq k \leq 13$<br>$-14 \leq l \leq 14$
Reflections collected	15405
Independent reflections	3469<br>R_{int} = 0.0196<br>R_{sigma} = 0.0170
Completeness to θ = 25°	99.6 %
Data / Restraints / Parameters	3469/0/257
Goodness-of-fit on F^2	1.027
Final R indexes [$I \geq 2\sigma(I)$]	R_1 = 0.0414<br>wR_2 = 0.1067
Final R indexes [all data]	R_1 = 0.0590<br>wR_2 = 0.1181
Largest peak/hole [eÅ^{-3}]	0.14/-0.17

3 Visualization of the crystal structure and molecular structure for ovatifolin

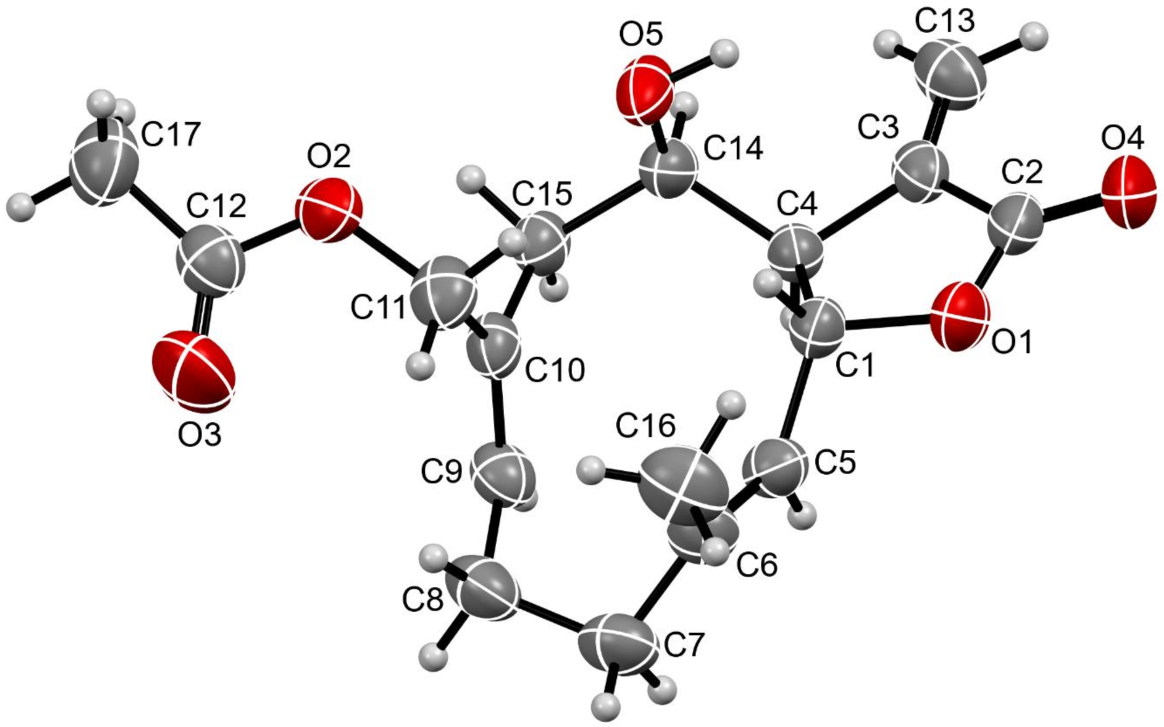

Figure S1: Molecular structure of ovatifolin with atomic labels. Displacement ellipsoids are shown at the 50% probability level.

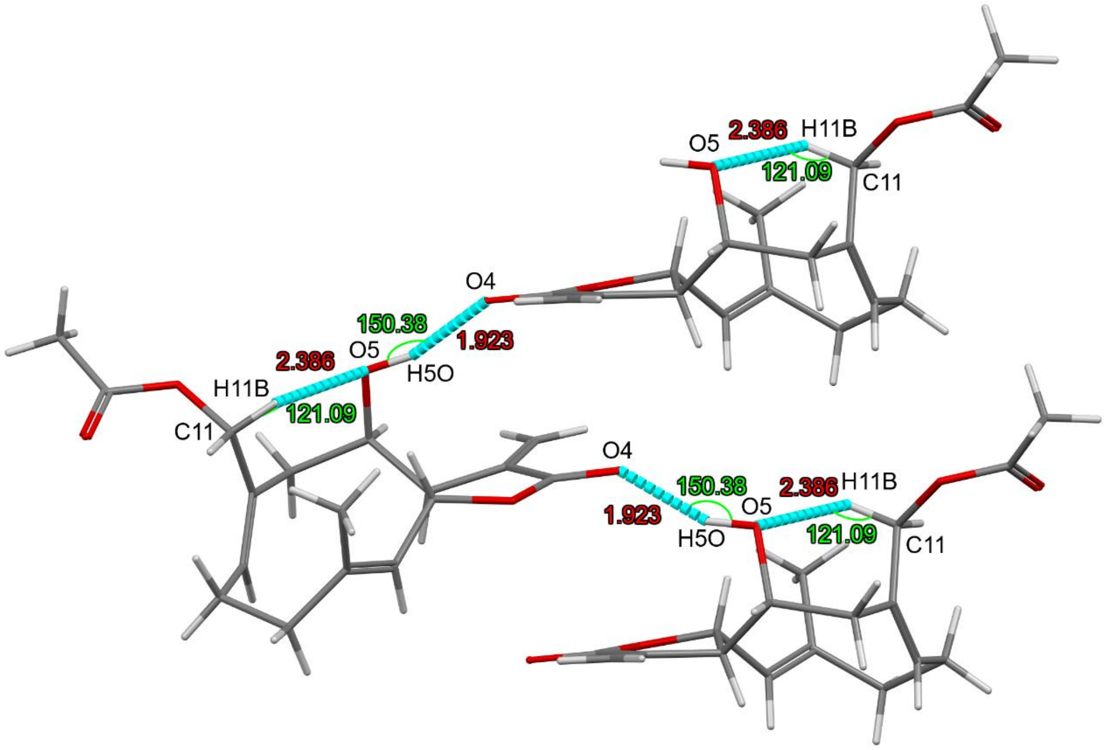

Figure S2: All hydrogen bonds in ovatifolin with A-H distances up to 2.6 Å (blue dashed lines) and D-H-A angles (green label) of at least 120°.

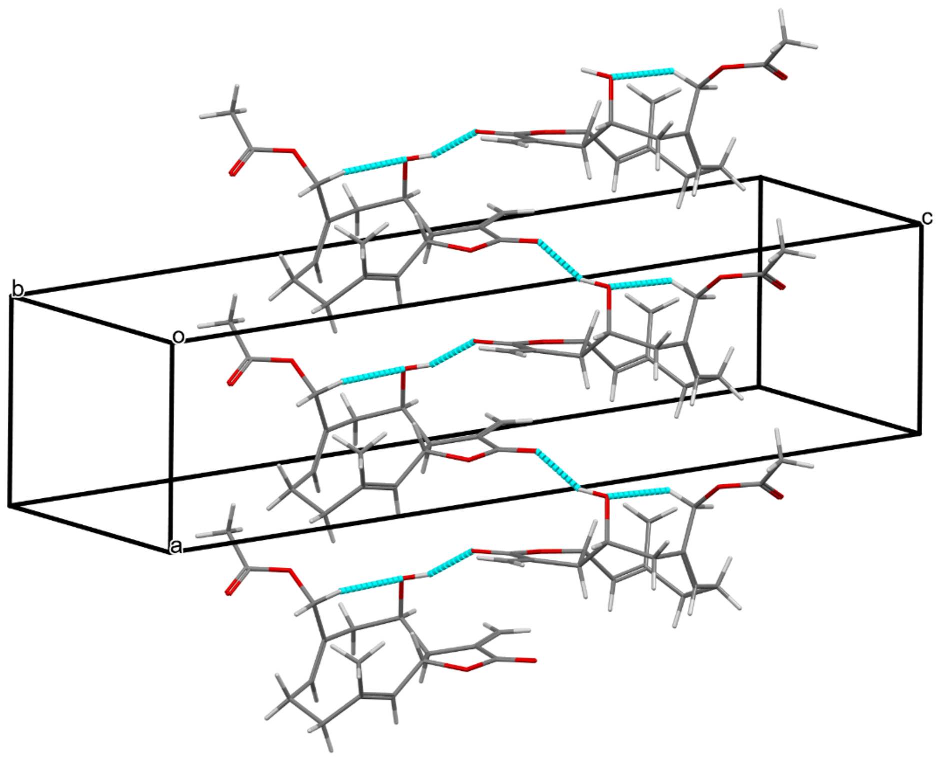

Figure S3: Zigzag-shaped hydrogen bonding sequence (blue dashed lines) along the a direction in ovatifolin.

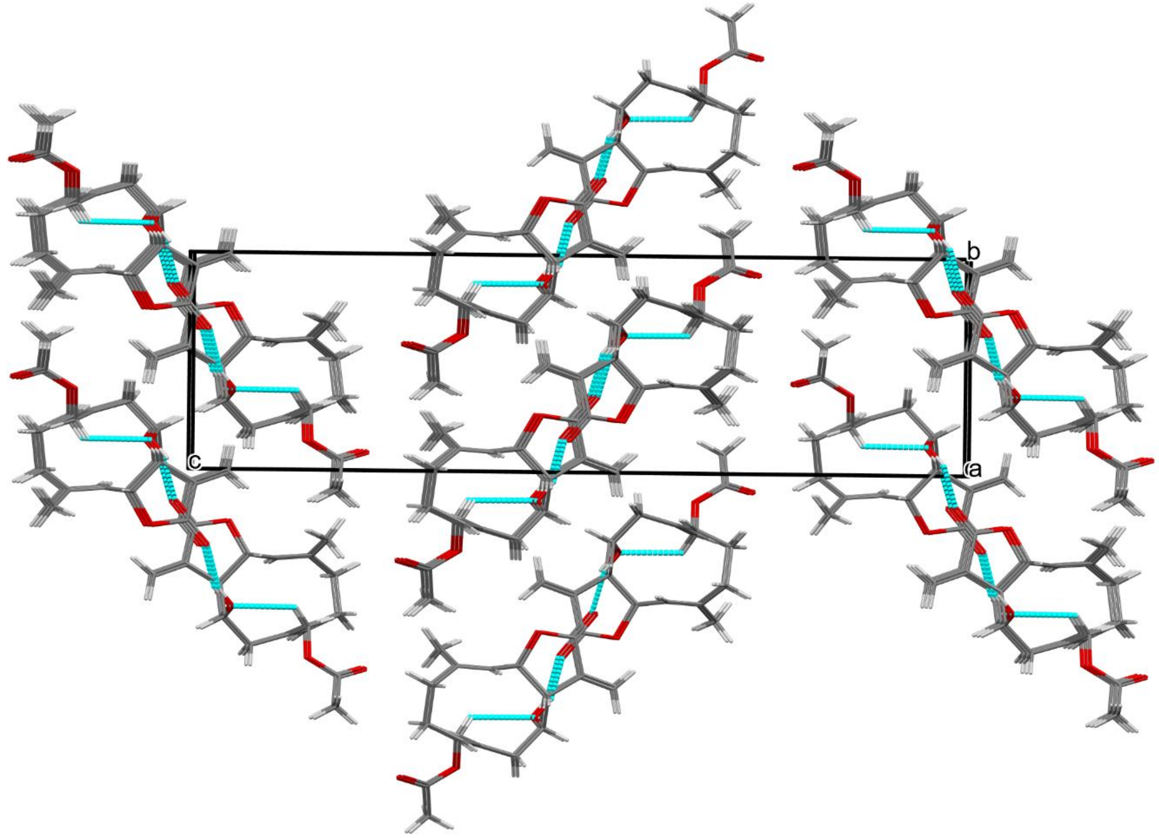

Figure S4: Packing of different hydrogen bonding chains (blue dashed lines) with view along the a direction in ovatifolin.

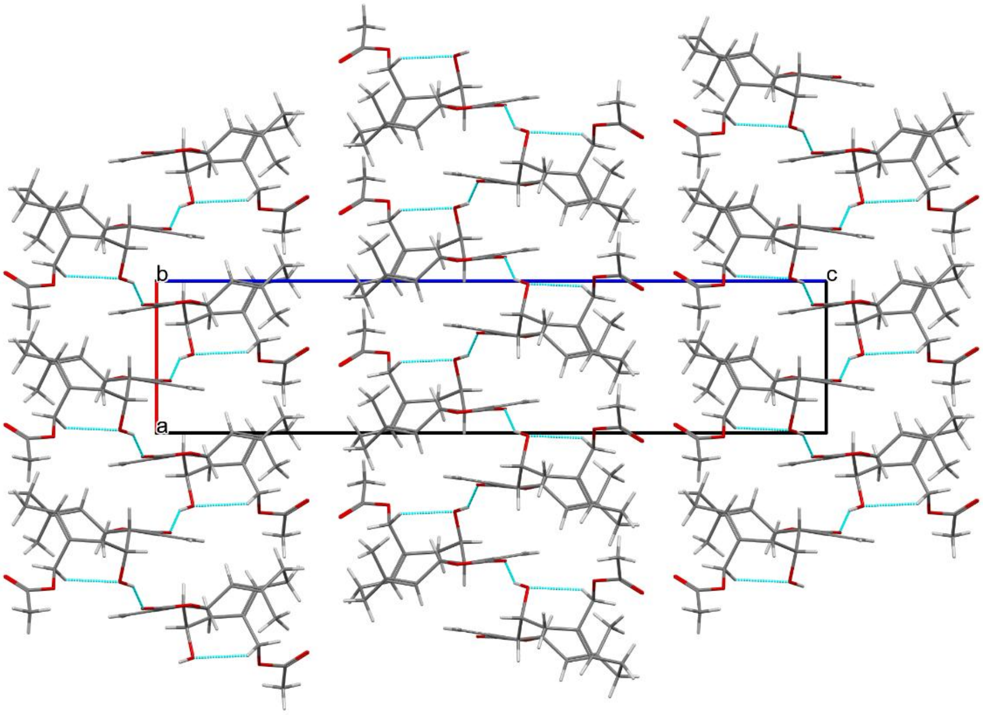

Figure S5: Cell view of ovatifolin looking along the crystallographic b axis (with hydrogen bonds as blue dashed lines).

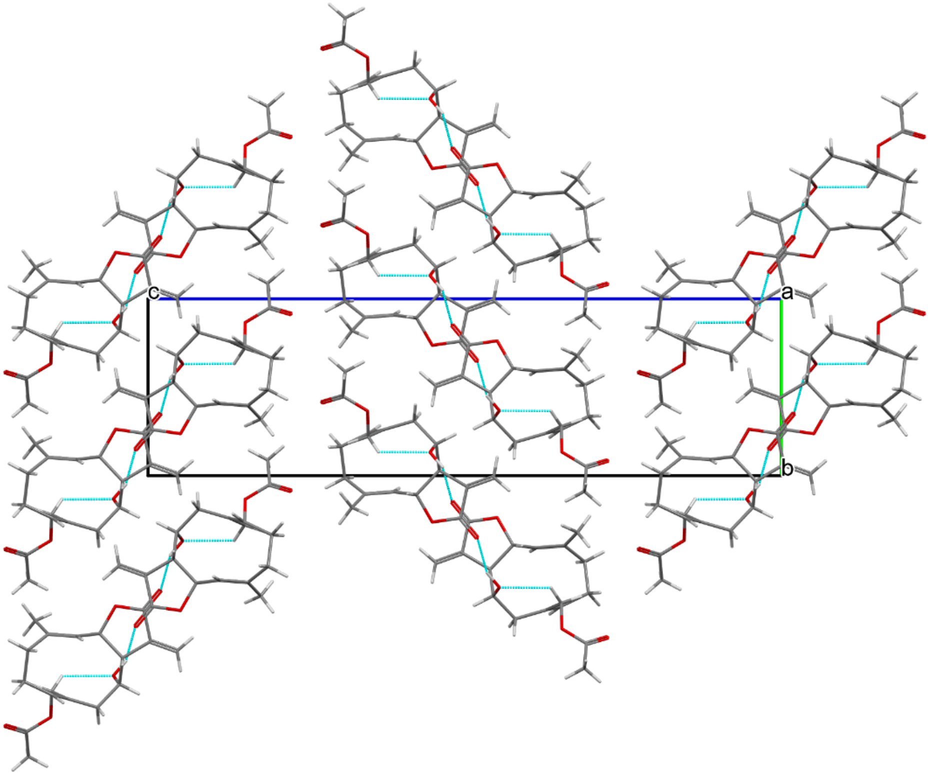

Figure S6: Cell view of ovatifolin looking along the crystallographic a axis (with hydrogen bonds as blue dashed lines).

B NMR-Spectroscopical analysis of ovatifolin

Table S2. NMR-data of ovatifolin and comparison with literature data[7] for ovatifolin.

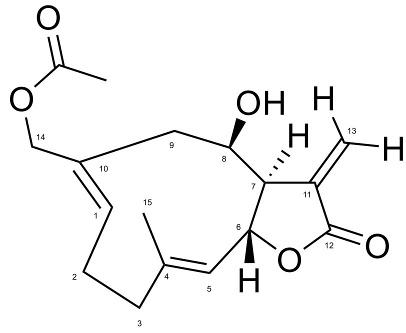

	This work[a]			Literature data for comparison[b]		
position	^{1}H in δ (ppm)	m (*J* (Hz))	^{13}C in δ (ppm)	^{1}H in δ (ppm)	m (*J* (Hz)	^{13}C in δ (ppm)
1	5.12	dd (12.3, 4.5)	136.5	5.14	dd	--
2	2.42<br>2.29	ddd (12.4, 12.4, 5.3)<br>m	25.8	2.35-2.10	m	--
3	2.36<br>2.15	m<br>m	39.1	2.35-2.10	m	--
4	--	--	142.2	--	--	--
5	4.84	d (10.2)	127.8	4.85	d	--
6	5.21	dd (10.2, 8.6)	75.0	5.20	dd	--
7	2.75	dt (8.5, 3.5)	53.7	2.76	m	--
8	4.58	m	71.3	4.59		--
9	2.94	dd (14.4, 5.4)	42.4	2.94 (α-H)<br>2.20 (β-H)	dd<br>d	--
10	--	--	133.2	--	--	--
11	--	--	138.4	--	--	--
12	--	--	170.3	--	--	--
13	6.34 (*Z*-H13)<br>5.57 (*E*-H13)	dm (3.1)<br>d (3.1)	120.5	6.36<br>5.58	d<br>d	--
14	4.77<br>4.58	d (11.9)<br>d (11.9)	63.2	4.80<br>4.58	d<br>d	--
15	1.62	d (1.3)	17.0	1.63	s	--
–*C*(O)CH$_3$	--	--	171.6	--	--	--
–C(O)*CH$_3$*	2.06	s	21.2	not reported	--	--

[a] ^{1}H-NMR (500 MHz, $CDCl_3$); ^{13}C-NMR (125 MHz, $CDCl_3$). [b] ^{1}H-NMR (270 MHz, $CDCl_3$); no ^{13}C-data were reported.

Figure S7: ^{1}H NMR (500 MHz, $CDCl_3$) of ovatifolin

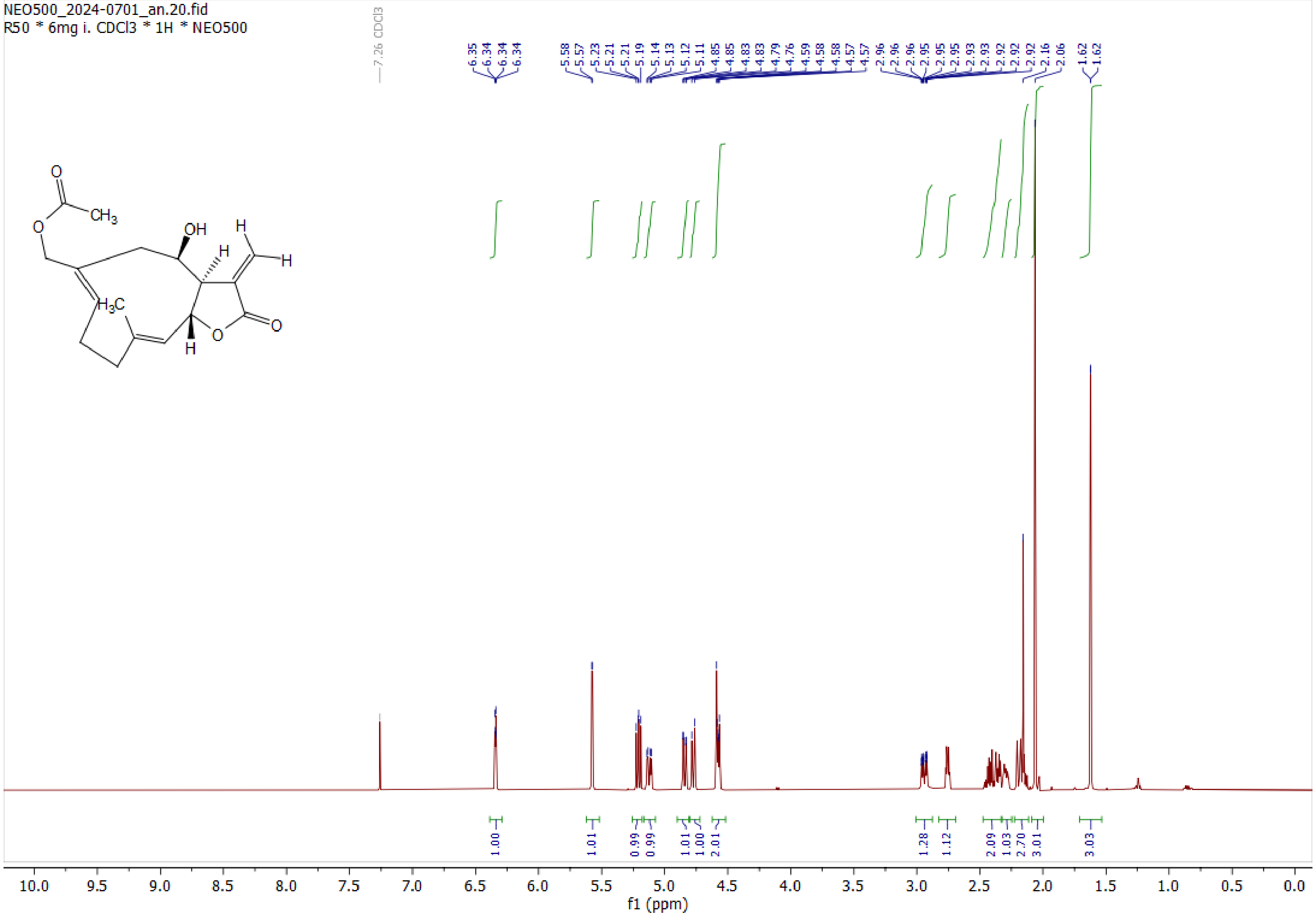

Figure S8: ^{13}C NMR (125 MHz, $CDCl_3$) of ovatifolin

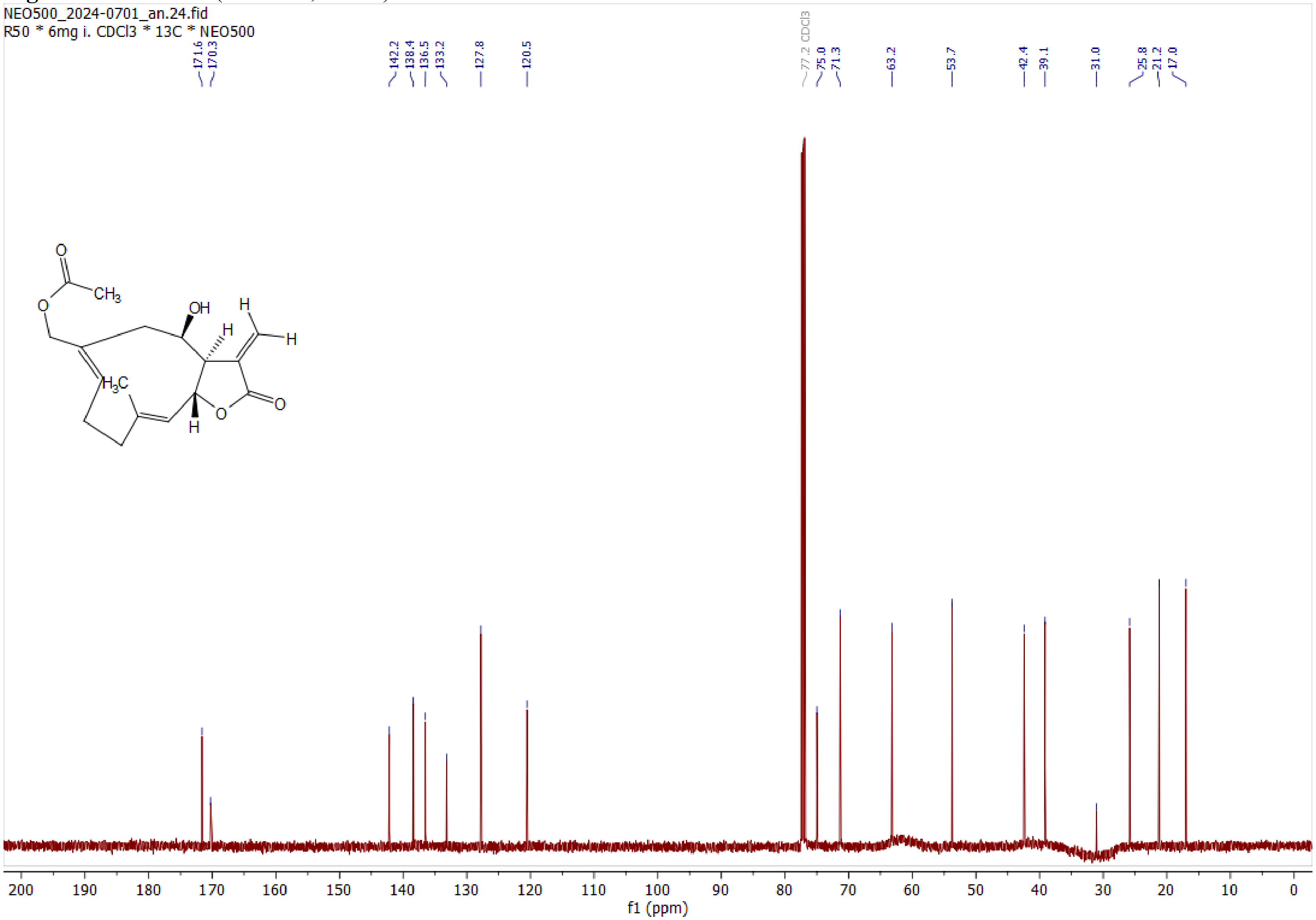

Figure S9: H,H-COSY (500 MHz, $CDCl_3$) of ovatifolin

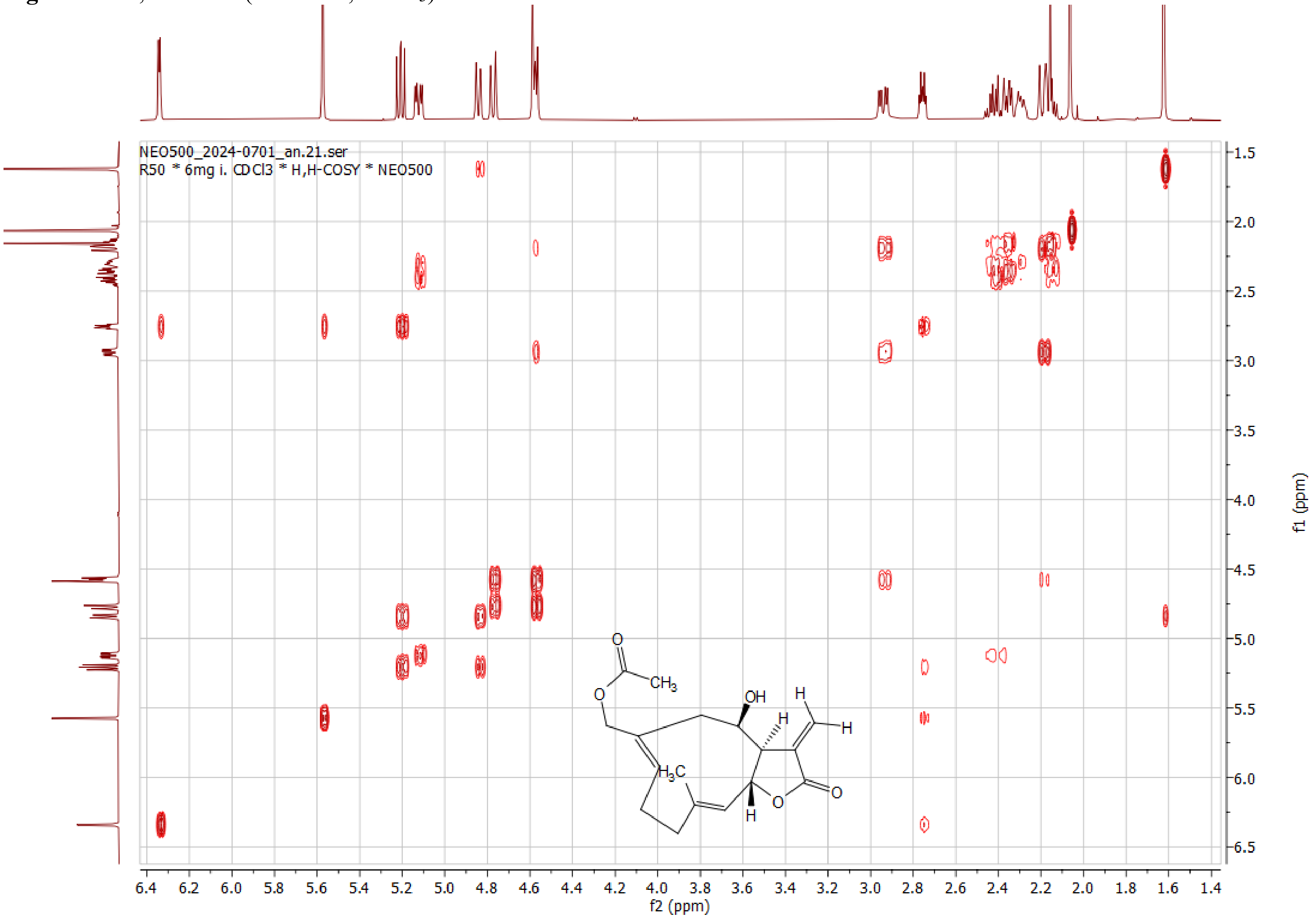

Figure S10: HSQC (500/125 MHz, $CDCl_3$) of ovatifolin

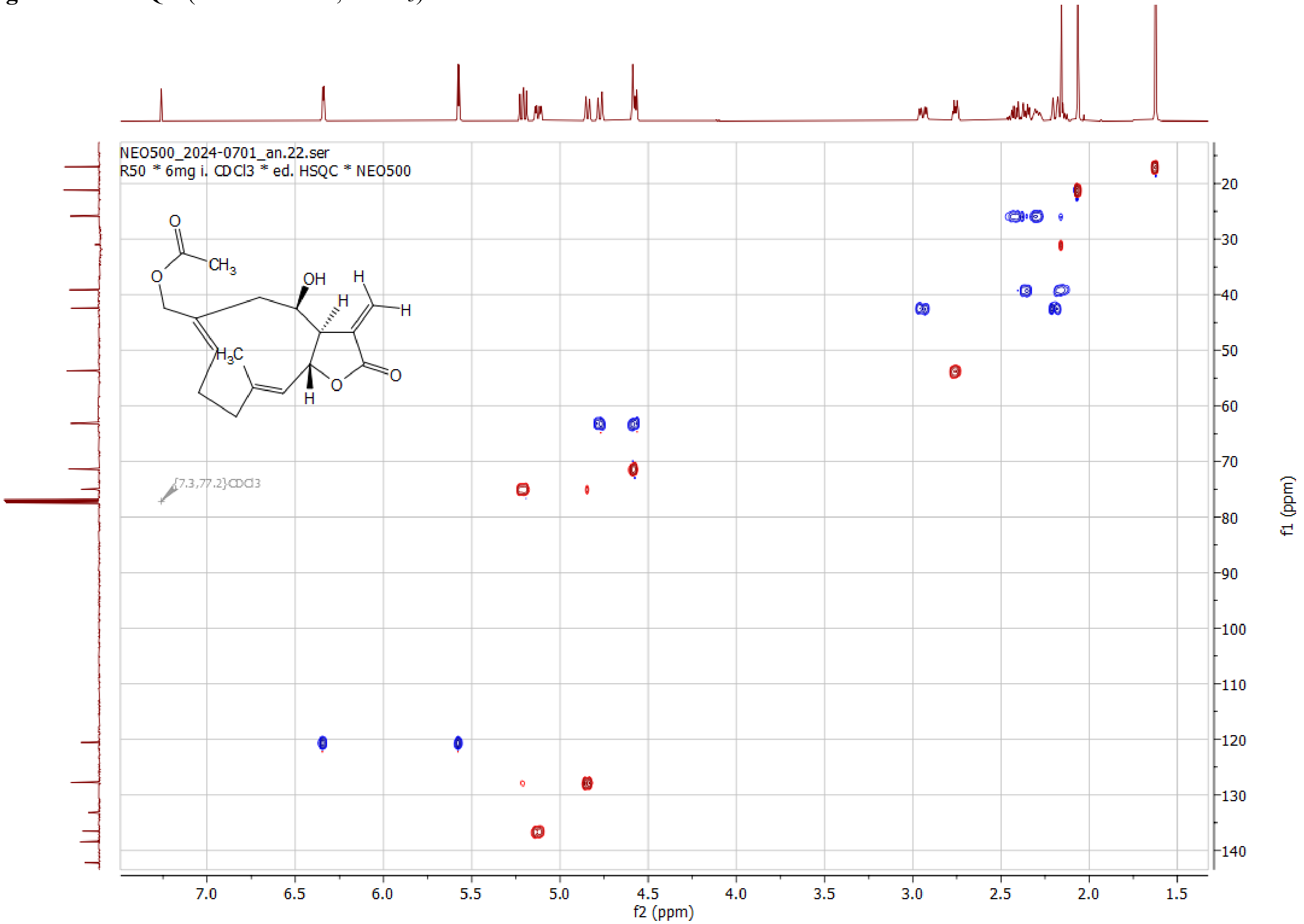

Figure S11: HMBC (500/125 MHz, $CDCl_3$) of ovatifolin

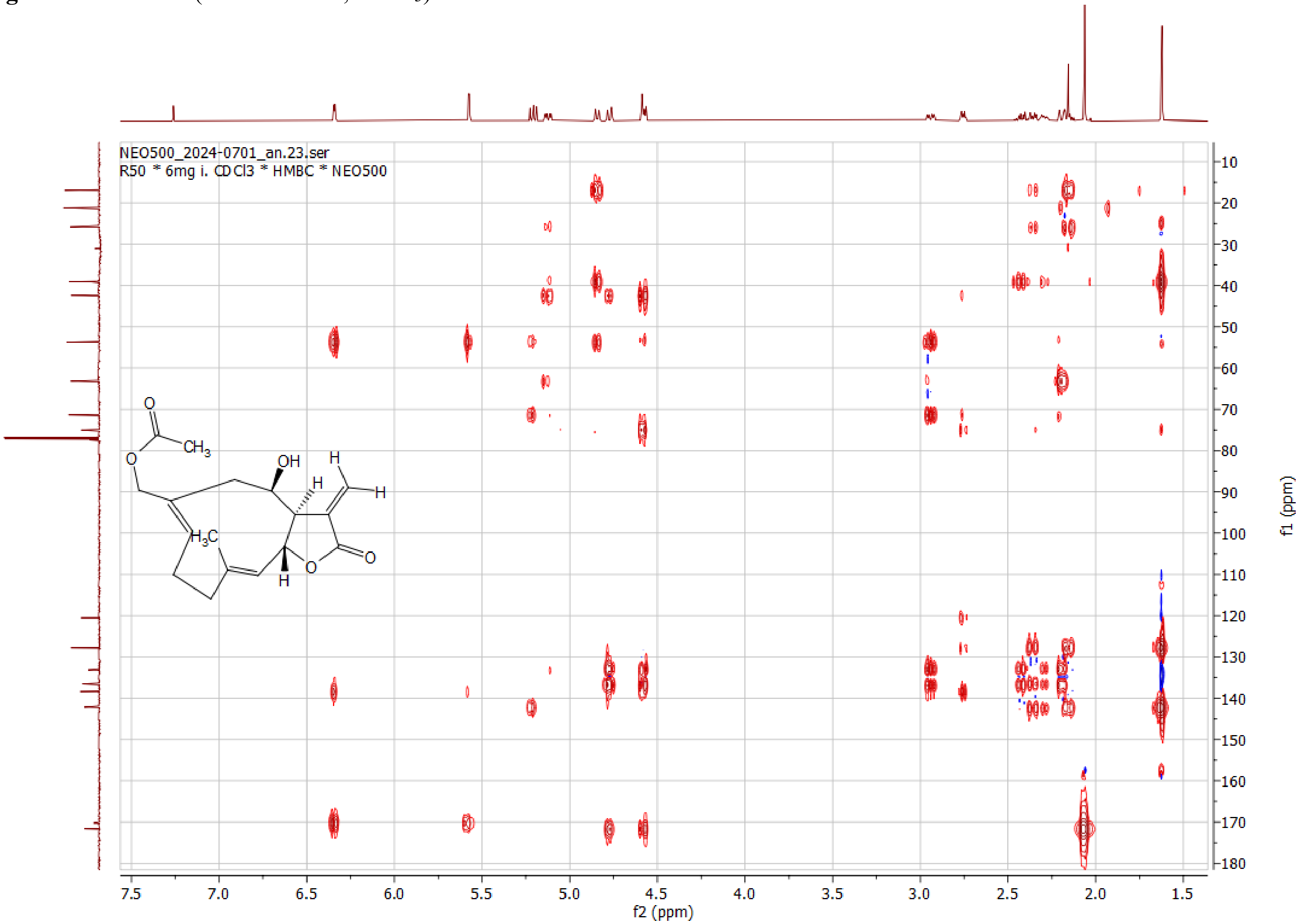

Supplement: Supplementary file 1 [file antioxidants-14-01392-s001.zip › antioxidants-3941430-supplementary.pdf]
